# Supplementary material for: ProteinShader: illustrative rendering of macromolecules
Source: BMC Struct Biol. 2009 Mar 30;9:19. doi: 10.1186/1472-6807-9-19 (PMC2672931; doi:10.1186/1472-6807-9-19)
Supplement: Additional file 1 — ProteinShader program without source code. This compressed file contains the complete ProteinShader program including associated libraries, but no source code. A README.txt file gives an overview of the ProteinShader distribution, and the index.html file in the help subdirectory has directions on getting started with the program as well as a set of tutorials. [file 1472-6807-9-19-S1.zip › ProteinShader-beta-0_9_4-binary/help/api/org/proteinshader/structure/enums/package-tree.html]

org.proteinshader.structure.enums Class Hierarchy (ProteinShader API)


|  |  |  |
| --- | --- | --- |
| **Overview** \| **Package** \| Class \| Use \| **Tree** \| **Deprecated** \| **Index** \| **Help** | |  |
| **PREV**   **NEXT** | **FRAMES**    **NO FRAMES**     **All Classes** |  |


---


## Hierarchy For Package org.proteinshader.structure.enums

**Package Hierarchies:**: All Packages

---

## Enum Hierarchy

- java.lang.**Object**
  - java.lang.**Enum**<E> (implements java.lang.Comparable<T>, java.io.Serializable)
    - org.proteinshader.structure.enums.**RegionEnum**- org.proteinshader.structure.enums.**HelixShapeEnum**- org.proteinshader.structure.enums.**ResidueEnum**- org.proteinshader.structure.enums.**DecorationEnum**- org.proteinshader.structure.enums.**AAColorEnum**- org.proteinshader.structure.enums.**HelixEnum**- org.proteinshader.structure.enums.**DrawableEnum**- org.proteinshader.structure.enums.**VisibilityEnum**- org.proteinshader.structure.enums.**BondEnum**- org.proteinshader.structure.enums.**SideChainEnum**- org.proteinshader.structure.enums.**AminoAcidEnum**- org.proteinshader.structure.enums.**AtomEnum**- org.proteinshader.structure.enums.**RegionColorEnum**- org.proteinshader.structure.enums.**CPKColorEnum**

---


|  |  |  |
| --- | --- | --- |
| **Overview** \| **Package** \| Class \| Use \| **Tree** \| **Deprecated** \| **Index** \| **Help** | |  |
| **PREV**   **NEXT** | **FRAMES**    **NO FRAMES**     **All Classes** |  |


---

# *Copyright © 2007-2008*
